# Sunflower centromeres consist of a centromere-specific LINE and a chromosome-specific tandem repeat

**DOI:** 10.3389/fpls.2015.00912

**Published:** 2015-10-31

**Authors:** Kiyotaka Nagaki, Keisuke Tanaka, Naoki Yamaji, Hisato Kobayashi, Minoru Murata

**Affiliations:** ^1^Applied Genomics Unit, Institute of Plant Science and Resources, Okayama UniversityKurashiki, Japan; ^2^NODAI Genome Research Center, Tokyo University of AgricultureSetagaya, Japan

**Keywords:** centromere, sunflower (*Helianthus annuus*), centromeric histone H3, centromeric DNA, ChIP-seq

## Abstract

The kinetochore is a protein complex including kinetochore-specific proteins that plays a role in chromatid segregation during mitosis and meiosis. The complex associates with centromeric DNA sequences that are usually species-specific. In plant species, tandem repeats including satellite DNA sequences and retrotransposons have been reported as centromeric DNA sequences. In this study on sunflowers, a cDNA-encoding centromere-specific histone H3 (CENH3) was isolated from a cDNA pool from a seedling, and an antibody was raised against a peptide synthesized from the deduced cDNA. The antibody specifically recognized the sunflower CENH3 (HaCENH3) and showed centromeric signals by immunostaining and immunohistochemical staining analysis. The antibody was also applied in chromatin immunoprecipitation (ChIP)-Seq to isolate centromeric DNA sequences and two different types of repetitive DNA sequences were identified. One was a long interspersed nuclear element (LINE)-like sequence, which showed centromere-specific signals on almost all chromosomes in sunflowers. This is the first report of a centromeric LINE sequence, suggesting possible centromere targeting ability. Another type of identified repetitive DNA was a tandem repeat sequence with a 187-bp unit that was found only on a pair of chromosomes. The HaCENH3 content of the tandem repeats was estimated to be much higher than that of the LINE, which implies centromere evolution from LINE-based centromeres to more stable tandem-repeat-based centromeres. In addition, the epigenetic status of the sunflower centromeres was investigated by immunohistochemical staining and ChIP, and it was found that centromeres were heterochromatic.

## Introduction

Asterales is the most diverged order of dicots and includes 11 families and 27,000 species. Among the 11 families, Asteraceae is the largest and includes the sunflower and daisy. Sunflowers (*Helianthus annuus* L., 2*n* = 2*x* = 34, genome size = 2.43 Gb/haploid) are one of the most important crops in Asterales because their seeds can be used for oil production (Bennett et al., 1982). Sunflowers have been genetically and cytogenetically investigated (Feng et al., 2013), and a genome sequencing project is now in progress (http://sunflowergenome.org/). For karyotypic analyses, repetitive DNA sequences including rDNA, sunflower-specific tandem repeats and bacterial artificial chromosome (BAC) clones have been used as probes, but none of these probes showed centromeric localization (Ceccarelli et al., 2007; Talia et al., 2010; Feng et al., 2013). In other words, at present, there is no genetic and cytogenetic marker for sunflower centromeres.

The kinetochore is a special protein complex formed on centromeric regions that ensures equal and accurate distribution of chromatids to daughter cells during mitosis and meiosis (Amor et al., 2004). Among the constitutive proteins of kinetochores, centromere-specific histone H3 (CENH3) acts as a base for assembling other kinetochore proteins, and its presence epigenetically determines the kinetochore position (Perpelescu and Fukagawa, 2011). The first identified CENH3 was CENP-A in humans (Earnshaw and Rothfield, 1985), and its orthologs have been isolated from 11 of 63 APG III orders, including Poales, Asparagales, Rosales, Fabales, Malpighiales, Malvales, Brassicales, Myrtales, Solanales, Asterales, and Apiales, in this decade (Talbert et al., 2002; Zhong et al., 2002; Nagaki et al., 2004, 2005, 2012b; Nagaki and Murata, 2005; Sanei et al., 2011; Wang et al., 2011; Neumann et al., 2012, 2015; Dunemann et al., 2014; Masonbrink et al., 2014; He et al., 2015; Maheshawari, 2015). However, no CENH3 has been isolated from Asterales species.

The CENH3s possess relatively conserved histone fold domains (HFDs) and highly variable N-terminal tails, and the HFD has been shown to be important for centromere localization (Vermaak et al., 2002; Black et al., 2004; Lermontova et al., 2006). The Loop 1 region on the HFD is longer than that of canonical histone H3, which is common among CENH3s. This region allows more compact packing of nucleosomes with CENH3 compared to those with canonical histone H3 (Black et al., 2004; Tek et al., 2010, 2011; Nagaki et al., 2012a; Maheshawari, 2015).

CENH3 is a component of core histones in the centromeric regions, and it directly binds to DNA. Additionally, CENH3 localizes to functional centromeres (Warburton et al., 1997; Nasuda et al., 2005; Han et al., 2006). Therefore, centromeric DNA sequences have been identified in plant species by chromatin immunoprecipitation (ChIP) using anti-CENH3 antibodies (Zhong et al., 2002; Nagaki et al., 2003, 2004, 2009, 2011, 2012a,b; Nagaki and Murata, 2005; Houben et al., 2007; Tek et al., 2010, 2011; Wang et al., 2011; Gong et al., 2012; Neumann et al., 2012; He et al., 2015). In most cases, species-specific microsatellites, minisatellites, macrosatellites, and retrotransposons have been identified as the centromeric sequences, and these sequences are located on all centromeric regions in the species (Zhong et al., 2002; Nagaki et al., 2003, 2009, 2011; Nagaki and Murata, 2005; Houben et al., 2007; Tek et al., 2010, 2011; Wang et al., 2011; Neumann et al., 2012; He et al., 2015).

Histone modifications are the key components in epigenetics, and they have been much investigated in this decade (Desvoyes et al., 2014; Sharma et al., 2015). In plant species, two different types of heterochromatin distribution are reported to be related to the genome size of the species (Houben et al., 2003). In plant species with a genome size smaller than 510 Mb, chromocenters appear on interphase nuclei, and heterochromatic modifications occur specifically on the chromocenters. *A. thaliana* is a good example for small-genome species, as it shows heterochromatic modifications on centromeric and pericentromeric regions not only at interphase but also at metaphase. Euchromatic modifications of the species were observed in the regions with no heterochromatic modifications (Houben et al., 2003; Jasencakova et al., 2003). In contrast, no chromocenters appear on interphase nuclei in plant species with genomes larger than 530 Mb. However, in these species, heterochromatic and euchromatic modifications are observed to disperse on interphase nuclei (Houben et al., 2003), and neither modification appears on centromeric and pericentromeric regions during metaphase.

The epigenetic modifications around centromeric regions have also been analyzed by ChIP using centromeric DNA sequences. In human cells, the centromere-specific histone H3 variants (CENP-A) coexist with heterochromatic modified histones at almost all stages during the cell cycle, but the histones are instantaneously modified with a euchromatic modification from anaphase to early G1 (Ohzeki et al., 2012). In rice, a CENH3-binding region in a centromere, Cen8, was revealed by ChIP to be heterochromatic (Nagaki et al., 2004). However, euchromatic markers were also detected in genic regions and 167-bp CentO variants in rice centromeres (Yan et al., 2006; Zhang et al., 2013).

In this study, we isolated a cDNA encoding CENH3 from sunflowers and raised a peptide antibody against CENH3. The antibody showed centromere-specific signals in immunostaining and immunohistochemical staining experiments. A ChIP-Seq experiment was also conducted to isolate DNA sequences that coexist with CENH3. Additionally, the epigenetic status of the centromeres was successfully revealed by immunohistochemical staining using anti-CENH3 and anti-modified histone antibodies as well as by ChIP-qPCR using these antibodies and isolated DNA sequences from ChIP-Seq.

## Materials and methods

### Plant material

Sunflower seeds (*H. annuus* L., 2*n* = 2*x* = 34, number 1802-065684) were obtained from a commercial source (LIC, Okayama, Japan).

### Identification of a sunflower expressed sequence tag (EST) encoding CENH3

An EST sequence encoding sunflower CENH3 (HaCENH3) was identified from the gene indices using the tblastn program (http://compbio.dfci.harvard.edu/tgi/) and the amino acid sequence of NtCENH3-1 (GenBank accession number: BAH03514, Nagaki et al., 2009) as a query.

### RNA isolation and PCR

Total RNA was isolated from a 3-day-old sunflower seedling using the RNeasy Plant Mini kit (Qiagen, Hilden, Germany). To determine the full-length cDNA sequence of a sunflower CENH3 gene, rapid amplification of cDNA ends (RACE) was conducted. For 3′RACE, the primer HaCENH3-3RACE (Supplementary Table 1) was designed from an EST encoding a putative sunflower CENH3 sequence found during the BLAST search; this primer was used with the SMARTer RACE cDNA Amplification Kit (Clontech, CA, USA). Another primer, HaCENH3-5RACE (Supplementary Table 1), was designed from the sequences determined by 3′RACE and was used to determine the 5′ end.

### Sequencing and sequence analyses

The 3′- and 5′-RACE products were cloned into a pGEM-T easy vector (Promega, WI, USA) and sequenced from both ends using a BigDye Terminator v1.1 cycle sequencing kit and an ABI PRISM 3130xl genetic analyzer (Applied Biosystems, CA, USA). A putative amino acid sequence was deduced from the DNA sequences and used as a query sequence for a protein BLAST search on the NCBI website (http://blast.ncbi.nlm.nih.gov/Blast.cgi?CMD=Web&PAGE_TYPE=BlastHome). The deduced HaCENH3 amino acid sequence was aligned with orthologs identified by the protein BLAST search and canonical histone H3 of rice using the Clustal X software program (Thompson et al., 1997). Phylogenetic relationships among the CENH3s were analyzed by the neighbor-joining method (Saitou and Nei, 1987).

### Immunostaining

Based on the deduced HaCENH3 amino acid sequence, a peptide corresponding to the N-terminus of HaCENH3 (H_2_N-ARTKHPAKRSSGIPADGRSS-COOH) was synthesized and injected into two rabbits. The raised antisera were purified using an affinity Sepharose column consisting of the aforementioned peptide.

Immunostaining was conducted as previously described (Nagaki et al., 2012b). In brief, root tips of 3-day-old sunflowers were fixed in microtubule stabilizing buffer (50 mM PIPES, pH 6.9, 5 mM MgSO4, and 5 mM EGTA) containing 3% (w/v) paraformaldehyde and 0.2% (v/v) Triton X-100. The fixed tips were washed and digested with a mixture of 1% (w/v) cellulase Onozuka RS (Yakult Pharmaceutical Industry, Tokyo, Japan) and 0.5% (w/v) pectolyase Y-23 (Seishin Pharmaceuticals, Tokyo, Japan) and then compressed onto slides coated with poly-L-lysine (Matsunami, Osaka, Japan). A 1:100 dilution of the purified anti-HaCENH3 antibody and monoclonal anti-modified-histone antibodies produced in mice (anti-histone H3 dimethyl K4 (H3K4me2): MBL (Nagoya, Japan) MABI0303 and anti-histone H3 dimethyl K9 (H3K9me2): MBL MABI0317) were applied to the slides. To detect acetylations of histone H4, an anti-histone H4 acetyl (H4Ac) antibody raised in rabbits (Millipore, MA, USA: #06-598) was used. The antibodies were detected using 1:1000 dilutions of Alexa Fluor 555-labeled anti-rabbit antibodies (Molecular Probes, OR, USA) and Alexa Fluor 488-labeled anti-mouse antibodies (Molecular Probes), respectively. Chromosomes were counterstained with 0.1 μg/ml 4,6-diamino-2-phenylindole (DAPI). Immunosignals and stained chromosomes were captured using a chilled charge-coupled device (CCD) camera, AxioCam HR (Carl Zeiss, Oberkochen, Germany), and images were pseudo-colored and processed using AxioVision software (Carl Zeiss).

### Immunohistochemical staining

Immunohistochemical staining was conducted as described with minor modifications (Yamaji and Ma, 2007; Nagaki et al., 2012b). Three-day-old sunflower roots were fixed as described above for immunostaining, and the fixed roots were sectioned at 100-μm thickness using a microslicer (LinearSlicer PRO10; Dosaka EM). These sections were transferred onto slides and then macerated. For three-color detection, 1:100 dilutions of the purified anti-HaCENH3 rabbit antibody and anti-α-tubulin mouse antibody (Sigma, MO, USA: T6199) were applied to the slides. For four-color detection, 1:100 dilutions of the purified anti-HaCENH3 rabbit antibody, anti-α-tubulin rat antibody (Abcam, Cambridge, UK: ab64332) and monoclonal anti-H3K9me2 mouse antibody (MBL MABI0317) were applied to the slides. After washing in PBS, the primary antibodies were detected using 1:1000 diluted secondary antibodies, the Alexa Fluor 555-labeled anti-rabbit antibodies and the Alexa Fluor 488-labeled anti-mouse antibodies for the three-color detection and Alexa Fluor 647-labeled anti-rabbit antibodies (Molecular Probes), Alexa Fluor 488-labeled anti-rat antibodies (Molecular Probes), and Alexa Fluor 546-labeled anti-mouse antibodies (Molecular Probes) for the four-color detection. Then, nuclei and chromosomes were counterstained with DAPI. Immunosignals and stained chromosomes were observed with a laser-scanning confocal microscope (LSM700; Carl Zeiss). The obtained data were analyzed using AxioVision software.

### Chromatin immunoprecipitation (ChIP)

ChIP was performed as previously described (Nagaki et al., 2012b) with minor modifications using the anti-HaCENH3 antibody and the anti-modified histone antibody (anti-H3K9me2: MBL #MABI0317, anti-H3K4me2: MBL #MABI0303 and anti-H4Ac: Millipore #06-598). Nuclei were isolated from the leaves of 1-month-old sunflowers and then digested with micrococcal nuclease (Sigma) to produce chromatin. Following overnight incubation of the chromatin with the antibodies at 4°C, the antibodies were captured using Dynabeads Protein G (Invitrogen, CA, USA). For mock experiments, a normal rabbit serum was used instead of the antibodies. DNA was purified from the chromatin with the captured antibodies by phenol/chloroform extraction followed by ethanol precipitation.

### ChIP-Seq and repeatexplorer analysis

ChIP-Seq was conducted using precipitated DNA from the input and HaCENH3 fractions in the ChIP. Libraries were constructed using the NEBNext ChIP-Seq Library Prep Reagent Set for Illumina (New England Biolabs, MA, USA), and the libraries were read by MiSeq (Illumina, CA, USA) with the paired-end 2 × 300 bp protocol. Conversion of raw base-call data to sequence data in the fastq format, identification of reads derived from each sample by index sequences, and adapter trimming were performed using MiSeq reporter 2.3.32. The sequence data were analyzed by a similarity-based clustering program, RepeatExplorer (http://www.repeatexplorer.org) (Novák et al., 2010) with default parameters.

### qPCR

qPCR was conducted using SYBR Premix Ex Taq II (Tli RNaseH Plus) (Takara, Shiga, Japan) and primers for qPCR (Supplementary Table 1) with a StepOne instrument (Applied Biosystems). The primers were designed based on the sequences in the clusters of the RepeatExplorer analysis. The precipitated DNA in the ChIP experiment was used as a template, and the mock was used as a negative control. Relative enrichment (RE) was calculated by the following formula: RE = amount of the sequence in the antibody fraction/amount of the sequence in the mock. The qPCR results were assessed by Student's *t*-test.

### Fluorescence *in situ* hybridization (FISH)

Probes were amplified using primer sets designed based on the sequences in clusters of the RepeatExplorer analysis (Supplementary Table 1) using sunflower genomic DNA as a template. The amplified DNA was cloned into pGEM T-easy, and sequences were confirmed using a BigDye Terminator v1.1 cycle sequencing kit and an ABI PRISM 3130xl genetic analyzer. To detect nucleolar organizing regions, an 18S-5.8S-28SrDNA clone from wheat (pTa71) was used (Gerlach and Bedbrook, 1979). To characterize sunflower chromosomes, a reported 386-bp tandem repetitive sequence, HAG004N15 (Ceccarelli et al., 2007), was amplified using specific primers (Supplementary Table 1) and cloned. The sequence was confirmed as described above.

FISH analysis of mitotic chromosomes was performed as previously described (Nagaki et al., 2011). Chromosomes were prepared from the root tips of 3-day-old sunflowers. The plasmid DNA was labeled by nick translation using a DIG-Nick Translation Mix (Roche, Basel, Switzerland) or a Biotin-Nick Translation Mix (Roche). The digoxigenin- and biotin-labeled probes were visualized using rhodamine-conjugated anti-digoxigenin antibody (Roche) and Alexa Fluor 488-conjugated streptavidin (Molecular Probes), respectively.

## Results

### Isolation and sequence analyses of CENH3 in sunflowers

To identify the HaCENH3 gene, a BLAST search was conducted in a sunflower EST database of the gene index project using the amino acid sequence of NtCENH3-1 as a query. One EST group (TC48348) containing a 675-bp sequence showed 68% identity to the query sequence. However, because of low quality in the first 150 bp of the EST, no start codon was found in the sequence. To obtain a full-length cDNA of the gene, 5′- and 3′-RACE experiments were performed using sunflower seedling cDNA as a template. As a result, a putative full-length cDNA containing a 429-bp ORF that encodes 143 amino acids (GenBank accession number LC075743) was obtained.

The amino acid sequence deduced from the ORF showed similarity to the sequences of some other plants, with CENH3 from *Daucus muricatus* showing the highest similarity (70%). The amino acid sequence of HaCENH3 was aligned with those of CENH3s from other plant species and rice canonical histone H3 (Supplementary Image 1). The alignment indicated that the N-terminal amino acid at position 14–44 of HaCENH3 did not show any similarity to those of the other CENH3s or the canonical histone H3, and it also indicated that HaCENH3 possessed a longer loop 1 domain than canonical histone H3. The longer loop 1 is a feature of CENH3s.

**Figure 1:**
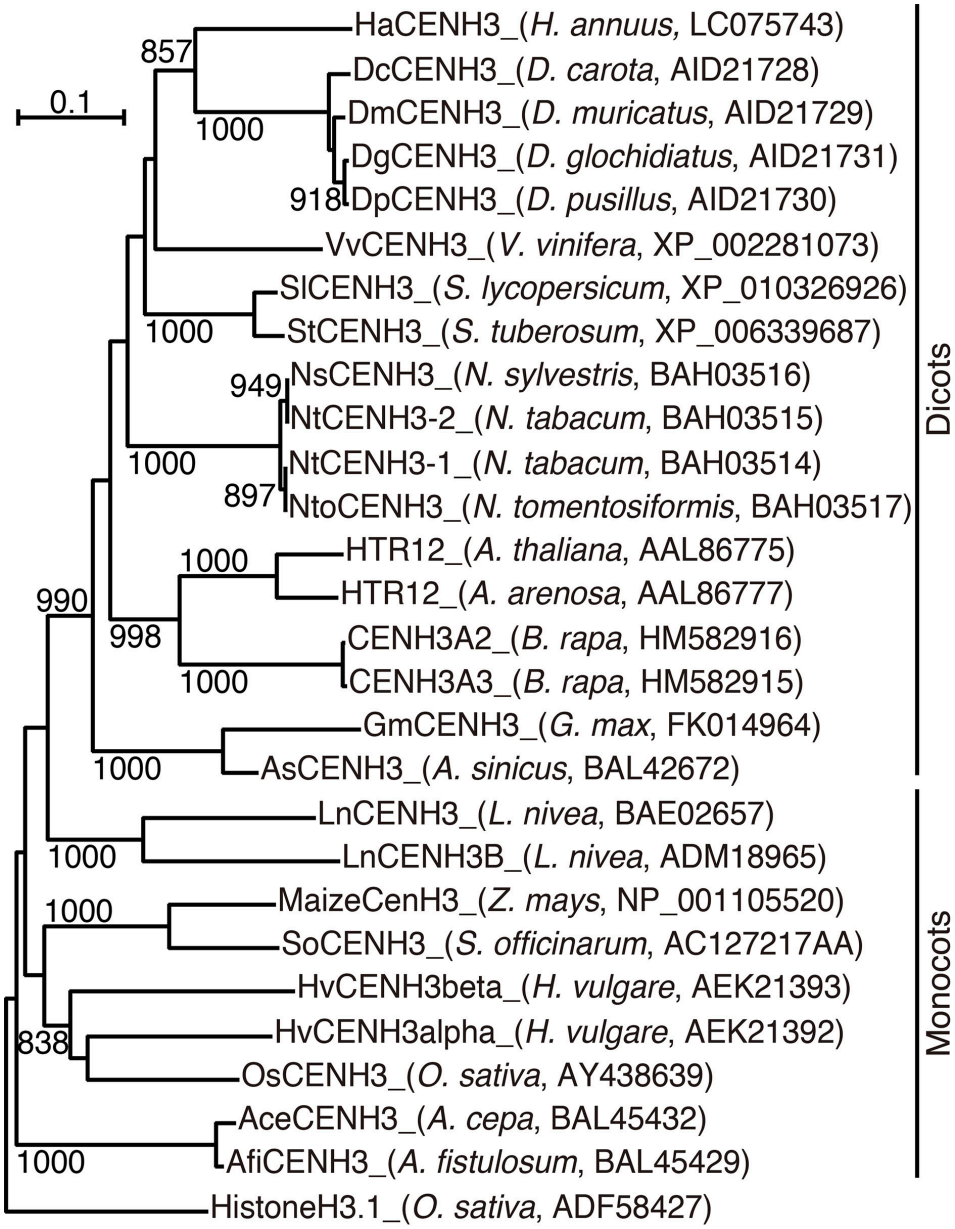
**Phylogenetic tree based on the amino acid sequences of plant CENH3**. The species name and GenBank accession number are indicated in parentheses. Rice canonical histone H3 was used as an outgroup. Bootstrap values greater than 800 in 1000 tests are indicated on the branches.

In a phylogenetic analysis using the alignment, HaCENH3 was classified into a dicot clade (Figure 1). As expected, HaCENH3 was found to be most closely related to the CENH3s of *Daucus* species, but the sequence was placed outside of a clade containing the *Daucus* CENH3s.

### Centromere localization of HaCENH3

**Figure 2:**
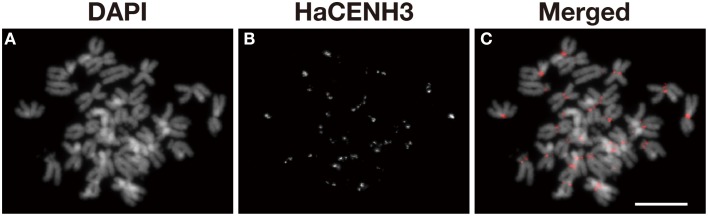
**Immunostaining of sunflower metaphase chromosomes using an anti-HaCENH3 antibody**. **(A)** DAPI-stained chromosomes. **(B)** Immunosignals of an anti-HaCENH3 antibody. **(C)** Merged image of **(A,B)**. Scale bar, 10 μm.

An anti-HaCENH3 antibody was raised against a synthetic peptide comprising N-terminal amino acid residues 2–21 of the deduced HaCENH3 amino acid sequence (Supplementary Image 1). To confirm its specificity to the centromeres, immunostaining using the antibody was conducted, and centromere-specific immunosignals appeared on all sunflower chromosomes (Figure 2). Additionally, in immunohistochemical staining, microtubule signals on all chromosomes were associated with all of the anti-HaCENH3 immunosignals at metaphase (Supplementary Movie 1).

### DNA sequences that interact with centromeric nucleosomes

To investigate DNA sequences that coexist with HaCENH3, a ChIP-Seq experiment was conducted using the anti-HaCENH3 antibody and chromatin extracted from sunflower leaves. DNA fragments from the input and the HaCENH3 fraction in the ChIP were sequenced using MiSeq with the paired-end 2 × 300 bp protocol and deposited in DDBJ (Accession number: DRA003719). After the initial quality checks, 1,795,002 paired reads from the input and 2,012,000 paired reads from the HaCENH3 fraction were analyzed using the RepeatExplorer program. By this analysis, a total of 372 clusters containing at least 0.01% of the used sequences were generated (Supplementary Data Sheet 1). Then, enrichment ratios (ERs) were calculated by the following formula: ER = HaCENH3 ChIP reads/input reads in the each cluster, and the clusters were sorted by ER (Supplementary Table 2). Out of the 372 clusters formed, 41 clusters showed an ER higher than 2.0.

**Figure 3:**
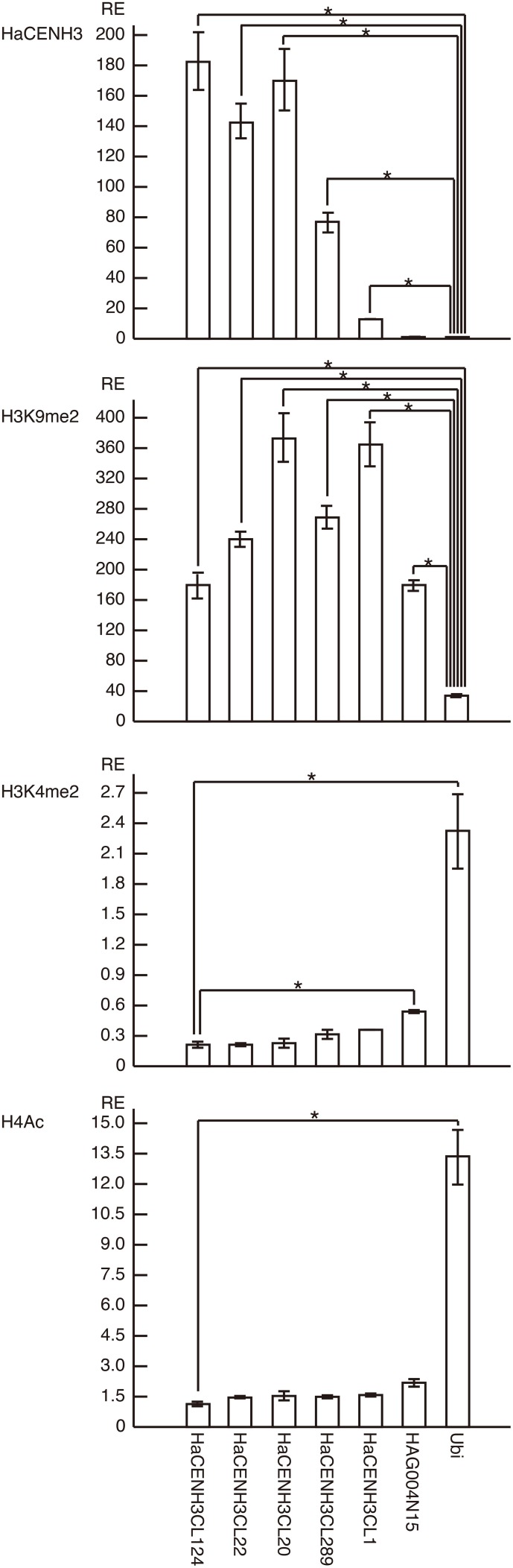
**ChIP-qPCR analysis of centromeric DNA sequences of sunflowers**. The columns and error bars represent the average relative enrichments (REs) and the standard errors from four independent ChIP reactions, respectively. The coding region of the sunflower ubiquitin gene (X14333) was used as a non-centromeric (negative) control in the anti-HaCENH3 ChIP and as a negative control in the anti-H3K9me2 ChIP. Since HaCENH3CL124 showed the lowest REs among the sequences in the anti-H3K4me2 and H4Ac ChIP, HaCENH3CL124 was used as a negative control. The statistical significance of differences between the negative controls and other sequences was determined using Student's *t*-test (^*^P < 0.01).

To confirm the ChIP-Seq results, ChIP-qPCR was conducted using the DNA sequences in five clusters (HaCENH3CL1, 20, 22, 124, and 289) selected from the 41 clusters having an ER higher than 2.0 (Figure 3). In the ChIP-qPCR, a sunflower ubiquitin gene (GenBank accession number: X14333) was used as a negative (non-centromeric) control. The DNA sequences from all five clusters were significantly increased (27-fold for HaCENH3CL1, 154-fold for HaCL289, 289-fold for HaCENH3CL22, 344-fold for HaCENH3CL22, and 370-fold for HaCENH3CL124) compared with the negative control in the CENH3 fractions (*P* < 0.01 via Student's *t*-test, *n* = 4), whereas a non-centromeric control sequence, HAG004N15, did not increase (*P* = 0.14 via Student's *t*-test, *n* = 4). Since the ER in the ChIP-Seq and the RE in the ChIP-qPCR showed a high correlation coefficient (*r* = 0.78) according to the Pearson product-moment correlation, the ChIP-qPCR data support the ChIP-Seq results. The significant increase in HaCENH3CL1 showing the minimum ER (2.0) among the five clusters in the ChIP-qPCR suggested that sequences in the 41 clusters having an ER higher than 2.0 in the ChIP-Seq coexisted with HaCENH3 in the sunflower genome.

The HaCENH3CL124 had the highest ER (52.0) and contained 187-bp tandem repeat sequences (Supplementary Table 2). The consensus DNA sequence of the tandem repeat was relatively AT-rich (58.3%), and no sequence similarity to DNA sequences in the GenBank database was found.

Of the 41 clusters, the program suggested that 25 clusters contained long interspersed nuclear element (LINE)-like sequences, and the sequences involve an ORF encoding an endonuclease (ENDO) and reverse transcriptase (RT) complex of LINE (Supplementary Table 2). These sequences show approximately 60% similarity to each other. Almost all RepeatExplorer cluster graphs of the LINE showed typical patterns of retrotransposons (line shape) rather than typical patterns of tandem repeats (star or ring shape) (Supplementary Image 2). As exceptional cases, two clusters, HaCENH3CL78 and HaCENH3CL115, involved 12 and four ENDO/RT-related reads, and showed star shape. Since RepeatExplorer splits some satellite repeats with long monomers, e.g., rDNA, into multiple clusters, connections of the LINE-like sequences clusters were investigated (Supplementary Table 3 and Supplementary Image 3). In the investigation, rDNA was used as a positive control of a satellite repeat with long monomers, and 17 clusters related to rDNA were found in the investigation. Ends of the rDNA clusters showed similarity to other clusters. On the other hand, almost all of the LINE clusters did not show frequent similarity hits observed among the rDNA clusters. As exceptional cases, two clusters, HaCENH3CL78 and HaCENH3CL115, showed frequent similarity hits to HaCENH3CL8, suggesting these are not LINE-like elements. Twenty-two of the remaining 23 LINE-like clusters showed ERs higher than 10.0. Additionally, junction of the elements and insertion sites were surveyed (Supplementary Image 4). If clusters include ends of mobile elements, junctions should be visible as sites with heterologous sequences at ends of contigs in the cluster. For example, a clear junction was observed in an end of HaCENH3CL189 showing a typical cluster graph of LTR-retrotransposon, and similar junctions were also observed in some of the LINE-like clusters. However, sunflower DNA sequences that were similar to the LINE-like sequences were not found in the GenBank DNA database. These results suggested that most centromeric regions of sunflowers are not involved in the current sunflower genome sequencing project because the project has not involved repetitive DNA sequence-rich regions (http://sunflowergenome.org/). An additional 12 of 41 clusters contained transposable element-like sequences, whereas DNA sequences in the remaining four clusters showed no homology to those registered in the GenBank DNA database.

**Figure 4:**
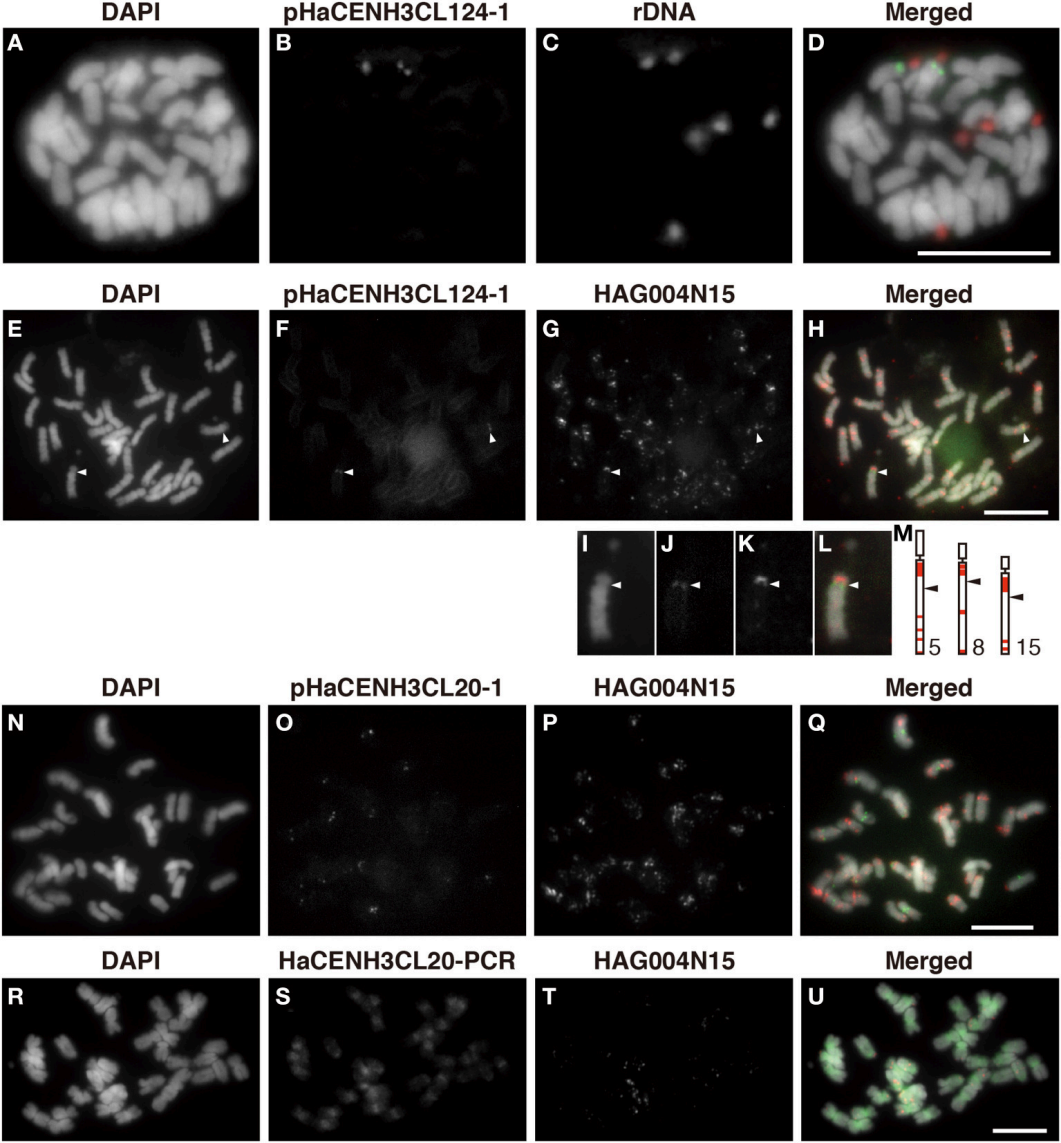
**FISH using the enriched sequence from ChIP-Seq**. **(A,E,I,N,R)** DAPI-stained sunflower chromosomes. **(B,F,J)** FISH signals of pHaCENH3CL124-1. **(C)** FISH signals of rDNA. **(G,K,P,T)** FISH signals of HAG004N15. **(O)** FISH signals of pHaCENH3CL20-1. **(S)** FISH signals of the PCR products of HaCENH3CL20. **(D)** A merged image of **(A–C)**. **(H)** A merged image of **(E–G)**. **(L)** A merged image of **(I–K)**. **(Q)** A merged image of **(N–P)**. **(U)** A merged image of **(R–T)**. **(I–L)** Enlarged images of **(E–H)**. **(M)** Karyograms from Ceccarelli's report (2007). White arrowheads in **(E–H)** and black arrowheads in **(M)** indicate centromeres on the HaCENH3CL124-positive chromosomes and the reported chromosomes, respectively. Scale bar, 10 μm.

To confirm the centromeric localization of the DNA sequences immunoprecipitated with anti-HaCENH3 in the ChIP-Seq and ChIP-qPCR, these sequences were used as probes in FISH analysis (Figure 4). A probe containing the HaCENH3CL124 sequence (pHaCENH3CL124-1, GenBank accession number: LC075744) showed centromeric signals on a pair of chromosomes with a secondary constriction (Figures 4A–D). To identify the chromosomes showing the HaCENH3CL124 signal, another FISH experiment in which the repeat and a reported FISH marker, HAG004N15, were used as probes was conducted; it revealed that the chromosome in question was chromosome 8 in Ceccarelli's report (Ceccarelli et al., 2007) (Figures 4E–M). A cloned probe containing the LINE-like sequence from HaCENH3CL20 (pHaCENH3CL20-1, GenBank accession number: LC075745) showed centromeric signals on almost all of the chromosomes (Figures 4N–Q). Since the LINE-like sequence showed centromeric signals, we named it HaCEN-LINE. In FISH using HaCENH3CL20-PCR products as probes, much stronger signals on all the centromeric and pericentromeric regions were found, but faint signals were also observed on the arm regions (Figures 4R–U). These results imply that the PCR products contain not only some HaCEN-LINE variants but also some non-centromeric LINE variants; centromeric variants are on all of the centromeres. Two Ty3-related DNA sequences from HaCENH3CL1 (pHaCENH3CL1-1, GenBank accession number: LC075747) and HaCENH3CL189 (pHaCENH3CL189-1, GenBank accession number: LC075746), when used as FISH probes, showed dispersed patterns with some strong spots on centromeres (Supplementary Image 5). These partial centromere localizations coincided with the partial enrichment of the sequences in the ChIP-Seq and ChIP-qPCR (Figure 3 and Supplementary Table 2), suggesting that these localize on both centromeric and non-centromeric arm regions.

### Epigenetic status of sunflower centromeres

**Figure 5:**
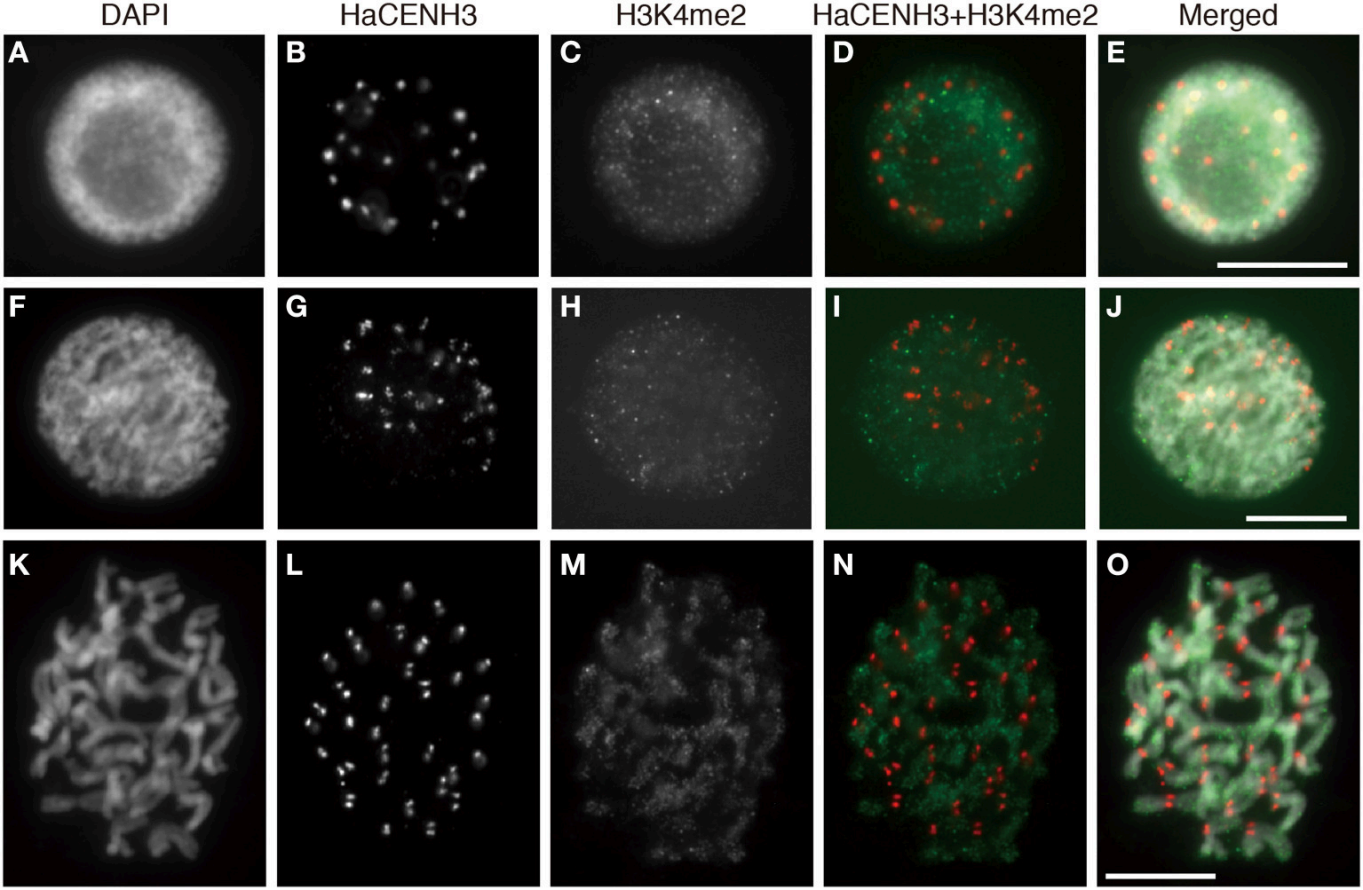
**Immunostaining using the anti-HaCENH3 and anti-H3K4me2 antibodies**. **(A–E)** An interphase nucleus. **(F–J)** Prophase chromosomes. **(K–O)** Metaphase chromosomes. Scale bar, 10 μm.

**Figure 6:**
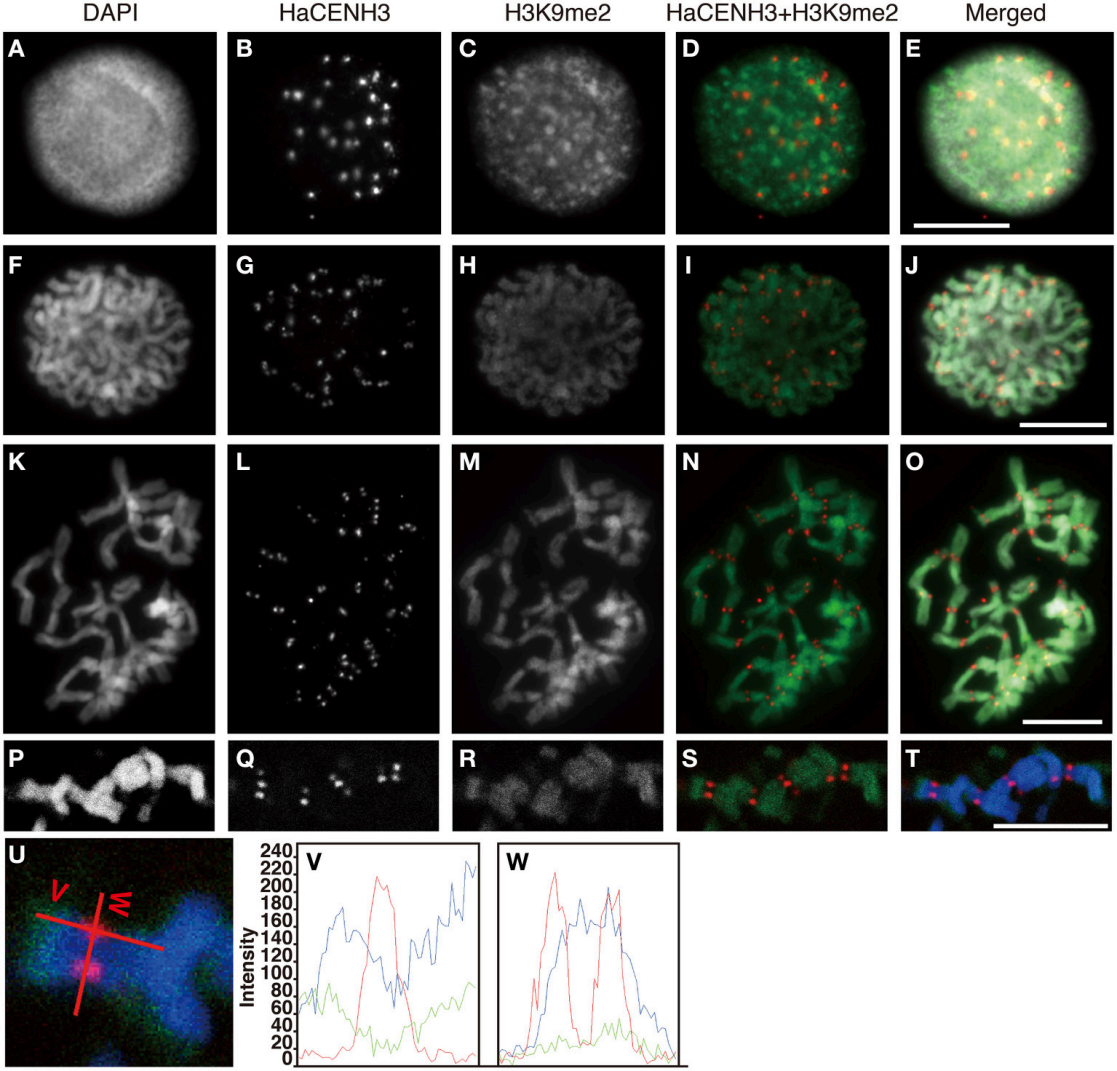
**Immunostaining and immunohistochemical staining using the anti-HaCENH3 and anti-H3K9me2 antibodies**. **(A–O)** Immunostaining images. **(A–E)** An interphase nucleus. **(F–J)** Prophase chromosomes. **(K–O)** Metaphase chromosomes. **(P–T)** Immunohistochemical staining images of metaphase chromosomes. Scale bar, 10 μm. **(U)** Scanning of metaphase chromosomes. Scanned positions are indicated as red lines. **(V)** A vertical scan of the chromosome. **(W)** A horizontal scan of the chromosome.

Post translational histone modifications of sunflower centromeres were investigated by immunostaining with four different antibodies against H3K4me2, H3K9me2, H3K9Ac, and H4Ac (Figures 5, 6 and Supplementary Image 6). In the interphase cells, HaCENH3 signals scattered as dots on nuclei, and polar organization, Rabl orientation, and chromocenters were not observed (Figures 5A–E, 6A–E and Supplementary Images 6A–E). H3K4me2, one of the representative euchromatic modifications, was detected on almost all regions of nuclei with many small dot signals, but the dot signals did not overlap with the HaCENH3 signals (Figures 5A–E). Similarly, the H3K4me2 signals did not co-localize with the HaCENH3 signals at prophase or metaphase (Figures 5F–O). Another euchromatic modification, H3K9Ac, showed a similar tendency at interphase and prophase (Supplementary Images 6A–E), but almost all H3K9Ac signals disappeared at metaphase (Supplementary Images 6F–J). Additionally, another euchromatic modification, H4Ac, showed stronger signals on the arms of metaphase chromosomes, but the signals on the centromeric and pericentromeric regions were weaker than those on the arms (Supplementary Images 6K–M).

Immunosignals from H3K9me2 that represented heterochromatin status were distributed on an entire region of nuclei with dots in the interphase cells, but the dot sizes were larger than these of H3K4me2 (Figures 6A–E). Although the larger dot signals did not overlap with the HaCENH3 signals, the HaCENH3 signals were colocalized with faint H3K9me2 signals. In the prophase and metaphase cells, the H3K9me2 signals appeared mainly on the chromosome arms, whereas centromeres and pericentric regions of chromosomes showed faint signals for histone modification (Figures 6F–O). Co-localization of HaCENH3 and H3K9me2 was confirmed by immunohistochemical staining using a laser-scanning confocal microscope (Figures 6P–W). On metaphase chromosomes, the HaCENH3 signals were colocalized with weak H3K9me2 signals, and both arms showed strong H3K9me2 (Figures 6U,V). In a horizontal-scanning of centromeres on a pair of chromatids (Figures 6U,W), a DAPI-staining peak appeared at the middle of the paired chromatids, and HaCENH3 signals were detected on both sides of the paired chromatids. On the other hand, H3K9me2 showed no peaks in the scan, and weak H3K9me2 signals appeared constantly on the centromeric region.

Histone modifications on centromeric DNA sequences were also investigated by ChIP-qPCR (Figure 3). In the analyses, the ubiquitin gene was used as a negative control for a heterochromatin marker, H3K9me2, and as a positive control for two euchromatin markers, H3K4me2 and H4Ac. In ChIP-qPCR with the anti-H3K9me2 antibody, all five HaCENH3-positive sequences and a non-centromeric repetitive sequence (HAG004N15) significantly accumulated compared with the negative control (*P* < 0.01 via Student's *t*-test, *n* = 4, Figure 3). For modification of H3K4me2, HaCENH3CL124 showed the lowest RE (0.21), and the REs of the positive control (ubiquitin) and HAG004N15 were 2.32 and 0.54, respectively (Figure 3). These two sequences significantly accumulated compared with HaCENH3CL124 (*P* < 0.01 via Student's *t*-test, *n* = 4). Similarly, HaCENH3CL124 showed the lowest RE (1.14) for H4Ac, and only the positive control (ubiquitin, RE = 13.36) increased significantly (*P* = 0.003 via Student's *t*-test, *n* = 4) compared with HaCENH3CL124 (Figure 3). Since leaf cells usually do not divide, these data suggest that all HaCENH3-positive sequences were involved with heterochromatin at interphase.

## Discussion

In this study, a CENH3-encoding cDNA was identified in sunflower. Based on the amino acid sequence deduced from the cDNA sequence, a peptide corresponding to the N-terminal 20 amino acids was synthesized. An antibody against the synthesized peptide recognized centromeres on all sunflower chromosomes. Using this antibody, ChIP-Seq analysis was applied and succeeded in isolating centromeric DNA sequences from sunflowers. Additionally, the epigenetic status at the centromeric DNA sequences was investigated by ChIP-qPCR with antibodies against modified histones.

Usually, centromeric DNA sequences in plant species consist of species-specific tandem repeats and retrotransposons, and these sequences are located on all centromeric regions (Zhong et al., 2002; Nagaki et al., 2003, 2009, 2011; Nagaki and Murata, 2005; Houben et al., 2007; Tek et al., 2010, 2011; Wang et al., 2011; Neumann et al., 2012; He et al., 2015). In this study of sunflower, we identified two types of centromeric DNA sequences (Supplementary Table 2 and Figure 4). One was a LINE-like sequence, HaCEN-LINE, which showed centromeric signals on all of the chromosomes (Figures 4N–U). Although some tandem repeats evolved from retrotransposons were reported in potato (Gong et al., 2012), no centromere-specific LINE-like elements have been reported. The cluster graphs of HaCEN-LINEs in RepeatExplorer showed line shape (Supplementary Image 2), implying their retroelement form rather than modified tandem repeat form. Therefore, this is the first report describing a centromere-specific LINE. Although the centromere-targeting mechanisms of transposable elements remain unknown, the present study indicated that the LINE-like sequence can also target centromeric regions in the same manner as retrotransposons. Another centromeric DNA sequence in sunflowers was a 187-bp tandem repeat, which was located on a single pair of chromosomes (Figure 4). Such chromosome-specific centromeric DNA sequences have been reported in chickens (Shang et al., 2010), tobacco (Nagaki et al., 2012a), and potatoes (Gong et al., 2012); however, they are not very common. The enrichment of the tandem repeats in HaCENH3 ChIP was much higher than that of the LINE (Figure 3), suggesting that the tandem repeat may be more useful for building more stable centromeres than the LINE. As discussed previously for other species (Nagaki et al., 2004, 2012a; Shang et al., 2010; Gong et al., 2012), sunflower centromeres may also be undergoing centromeric DNA evolution to equalize centromeric DNA sequences among chromosomes; the tandem repeats may form stabilized centromeres on the all sunflower chromosomes after this evolutionary event.

With the exception of phosphorylation, other histone modifications on the centromeric regions are not well characterized in plants (Desvoyes et al., 2014; Sharma et al., 2015). According to Houben et al. (2003), plant species with a genome smaller than 510 Mb formed chromocenters at interphase, and H3K9me2 preferentially occurred on the chromocenters. In *Arabidopsis*, H3K9me2 signals appeared on all centromeric and pericentromeric regions of metaphase chromosomes. Euchromatin-specific histone modifications, such as H3K4me2 and H4Ac, were observed in an inverse pattern compared to that of the H3K9me2 (Houben et al., 2003; Jasencakova et al., 2003). In plant species with genomes larger than 530 Mb, such as barley, no chromocenters were observed, and dispersed H3K9me2 signals were observed at interphase; their low level of modification appears on centromeric and pericentromeric regions of metaphase chromosomes (Fuchs et al., 2006). Occasionally, H4Ac were detected at centromeric and pericentromeric regions of metaphase chromosomes in barley and field beans (Jasencakova et al., 2000, 2001). In this study, sunflowers showed dispersed modification patterns for H3K4me2, H3K9Ac and H3K9me2 at interphase and a lower level of H3K9me2 modification on metaphase centromeres (Figures 5, 6). However, the irregular modification patterns observed at metaphase in barley and field bean were not detected at metaphase in sunflower. The genome size of sunflower was estimated to be 2.43 Gb (Bennett et al., 1982), and the observed distribution patters of the modified histones in this study (Figures 5, 6 and Supplementary Image 6) coincided with those in plants with large genomes (Houben et al., 2003).

To determine histone modification events on each different centromeric DNA sequence, ChIP analysis has higher resolution and quantitative capacity than immunostaining. In rice, ChIP revealed that centromeric DNA sequences excepting the genic regions on chromosome 8 were heterochromatic (Nagaki et al., 2004; Yan et al., 2006). Subsequently, different histone modifications were observed in CentO sequences (Zhang et al., 2013). CentO sequences with regular size units (155 bp) were enriched in OsCENH3 ChIP, whereas 167-bp CentO variants were enriched in a euchromatic modification, H3K4me2, ChIP rather than OsCENH3. In the present study with sunflowers, two centromeric DNA sequences showed heterochromatic status (Figure 3), suggesting that the status of plant centromeres at interphase was heterochromatic. Additionally, immunohistochemical staining revealed the existence of H3K9me2 on the centromeres at metaphase (Figure 6). Furthermore, immunostaining showed that the level of H3K9Ac in the interphase nuclei was higher than that on metaphase chromosomes (Supplementary Image 6). Although we could not quantify the H3K9Ac level on centromeres, the increased level of H3K9Ac in the interphase nuclei implies that it increased on centromeres as well. In the case of human cells, the combination of rigid cell cycle control and ChIP made it possible to detect H3K9Ac within a short range of time during the cell cycle (Ohzeki et al., 2012). To investigate H3K9Ac and other modifications on plant centromeres, a more accurate quantification system, such as that utilized in human cells, is required in the future.

### Conflict of interest statement

The authors declare that the research was conducted in the absence of any commercial or financial relationships that could be construed as a potential conflict of interest.

## Supplementary material

Supplementary Image 1 **Amino acid sequence alignment of plant CENH3 and a canonical histone H3**. Species name and GenBank accession numbers are indicated in parentheses. A red box indicates the amino acid residues used for raising a peptide antibody against HaCENH3. A magenta box indicates the position of Loop 1.

Supplementary Image 2 **RepeatExplorer cluster graphs of the enriched sequence from ChIP-Seq**. RepeateExplorer cluster graphs were indicated with their cluster name. Repeat types are indicated by magenta (tandem repeat), orange (LINE), green (LTR-retrotransposon), and black (others). 5SrDNA and HAG004N15 are examples of tandem repeats.

Supplementary Image 3 **Connection of RepeatExplorer cluster graphs of rDNA and HaCEN-LINE**. Connection of RepeateExplorer cluster graphs were indicated with their cluster name.

Supplementary Image 4 **Junction of HaCEN-LINE and its insertion sites**. Junction of HaCEN-LINE and its insertion sites were indicated with their cluster name.

Supplementary Image 5 **FISH using the accumulated sequence from ChIP-Seq. (A,D)** DAPI-stained sunflower chromosomes. **(B)** FISH signals of pHaCENH3CL1-1. **(E)** FISH signals of pHaCENH3CL189-1. **(C)** A merged image of **(A,B)**. **(F)** A merged image of **(D,E)**. Scale bar, 10 μm.

Supplementary Image 6 **Immunostaining using the anti-HaCENH3, anti-H3K9Ac, and anti-H4Ac antibodies. (A–E)** An interphase nucleus (top) and prophase chromosomes (bottom). **(F–M)** Metaphase chromosomes. **(A,F,K)** DAPI staining. **(B,G)** Immunosignals of anti-HaCENH3. **(C,H)** Immunosignals of anti-H3K9Ac. **(L)** Immunosignals of anti-H4Ac. **(D)** A merged image of **(B,C)**. **(E)** A merged image of **(A–C)**. **(I)** A merged image of **(G,H)**. **(J)** A merged image of **(F–H)**. **(M)** A merged image of **(K,L)**. Scale bar, 10 μm.

Supplementary Movie 1 **A video including immunohistochemical images**. HaCENH3 (red), α-tubulin (green), and DAPI (gray).

Supplementary Data Sheet 1 **A FASTA file containing HaCENH3CLs**.

Supplementary Table 1 **Primers used in this study**.

Supplementary Table 2 **Clusters possessing an enrichment ratio (ER) higher than 2.0 in the RepeatExplorer analysis**.

Supplementary Table 3 **Similarity hits on rDNA and HaLINE clusters**.
