# Supplementary figures and images for: Sunflower centromeres consist of a centromere-specific LINE and a chromosome-specific tandem repeat
Source: Front Plant Sci. 2015 Oct 31;6:912. doi: 10.3389/fpls.2015.00912 (PMC4628103; doi:10.3389/fpls.2015.00912)

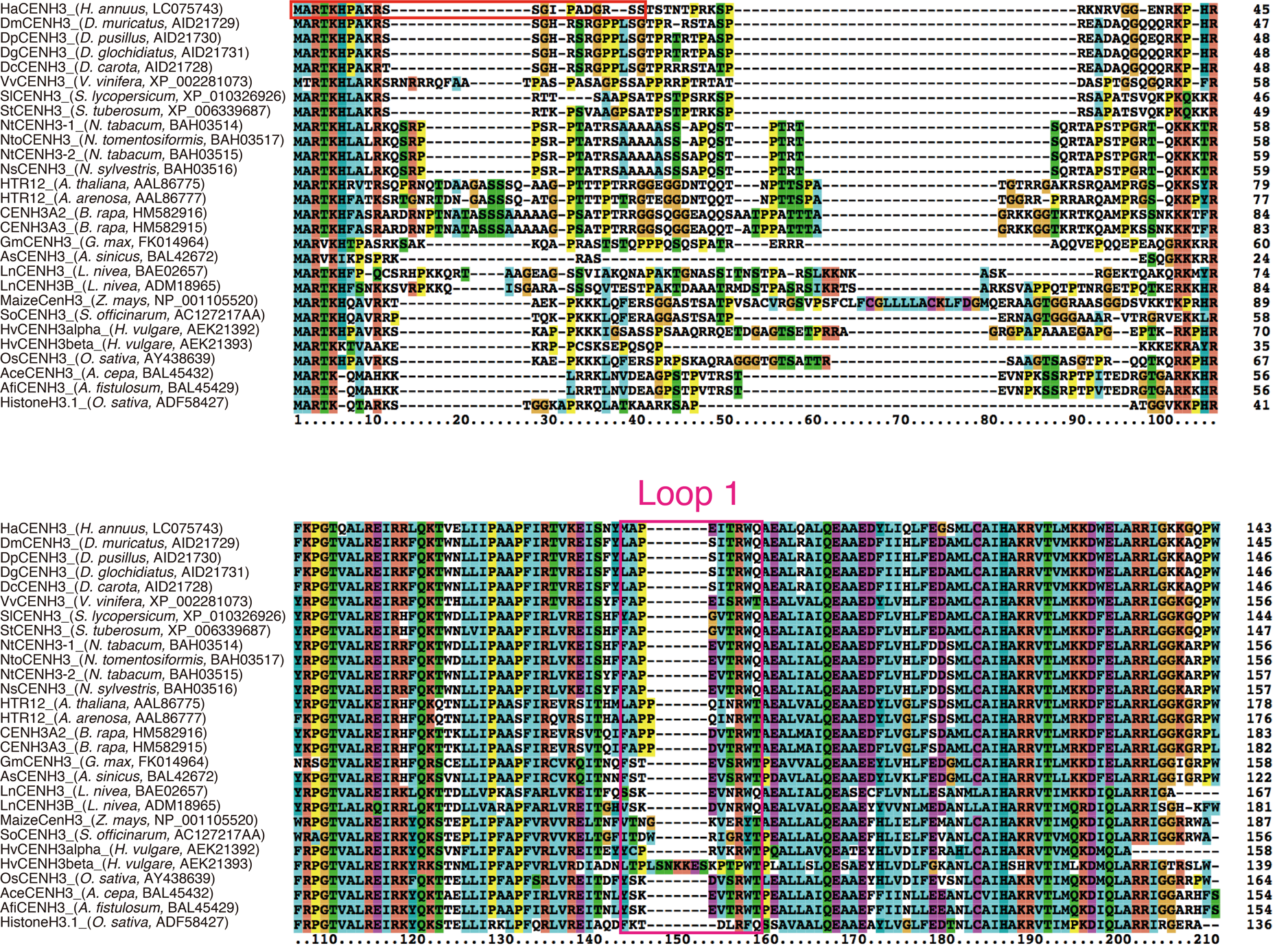

Supplement: Supplementary Image 1 — Amino acid sequence alignment of plant CENH3 and a canonical histone H3. Species name and GenBank accession numbers are indicated in parentheses. A red box indicates the amino acid residues used for raising a peptide antibody against HaCENH3. A magenta box indicates the position of Loop 1. [file Image1.TIF]

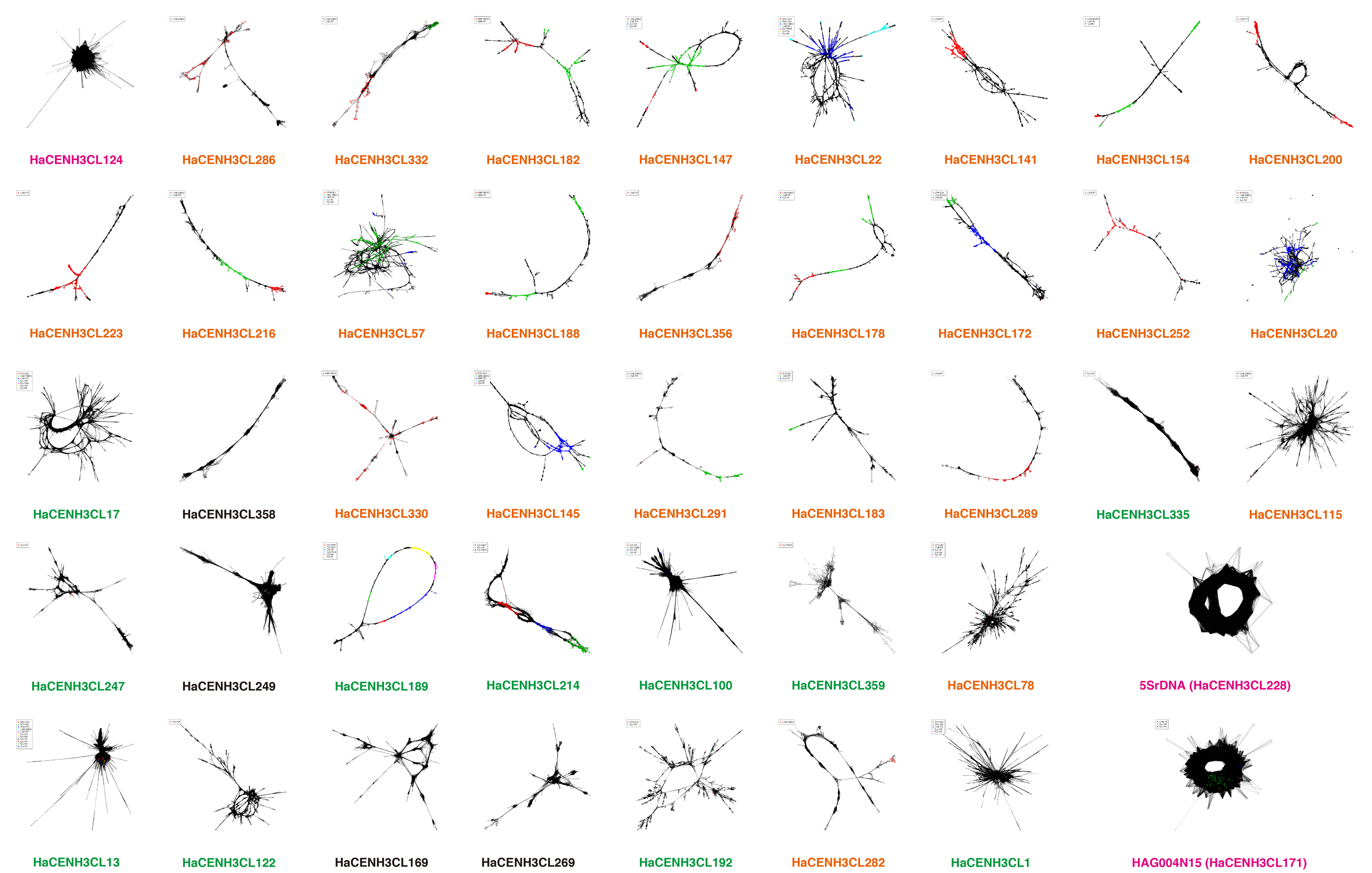

Supplement: Supplementary Image 2 — RepeatExplorer cluster graphs of the enriched sequence from ChIP-Seq. RepeateExplorer cluster graphs were indicated with their cluster name. Repeat types are indicated by magenta (tandem repeat), orange (LINE), green (LTR-retrotransposon), and black (others). 5SrDNA and HAG004N15 are examples of tandem repeats. [file Image2.TIF]

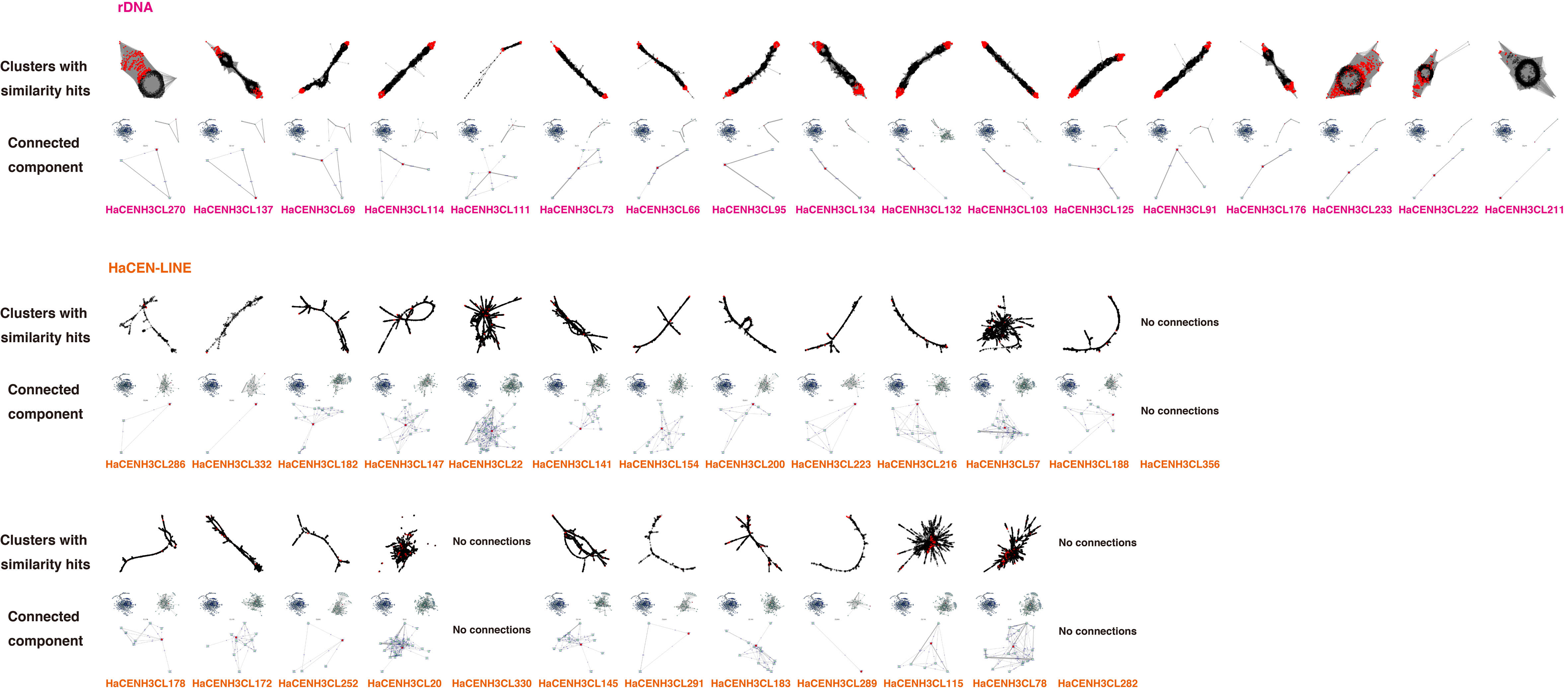

Supplement: Supplementary Image 3 — Connection of RepeatExplorer cluster graphs of rDNA and HaCEN-LINE. Connection of RepeateExplorer cluster graphs were indicated with their cluster name. [file Image3.TIF]

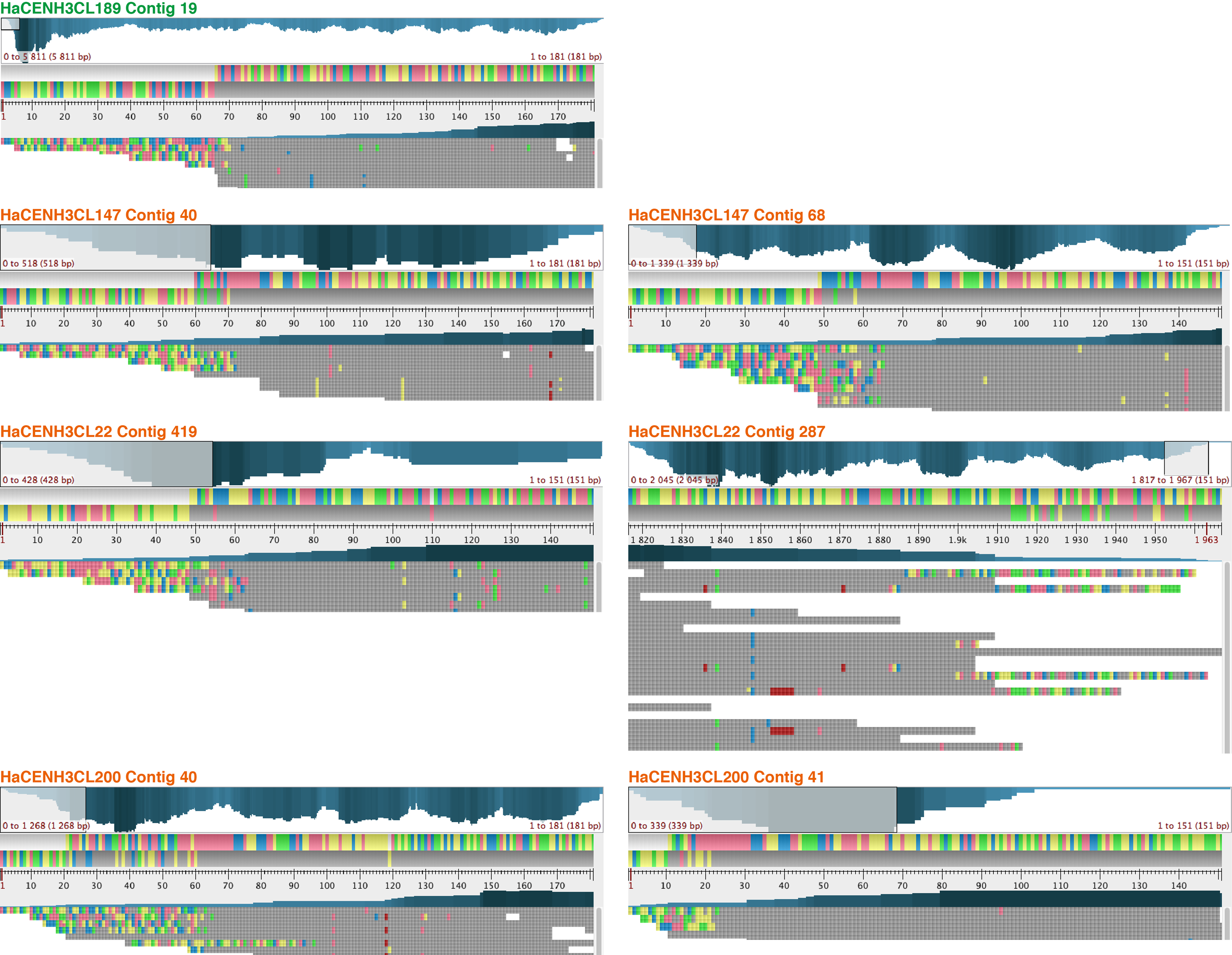

Supplement: Supplementary Image 4 — Junction of HaCEN-LINE and its insertion sites. Junction of HaCEN-LINE and its insertion sites were indicated with their cluster name. [file Image4.TIF]

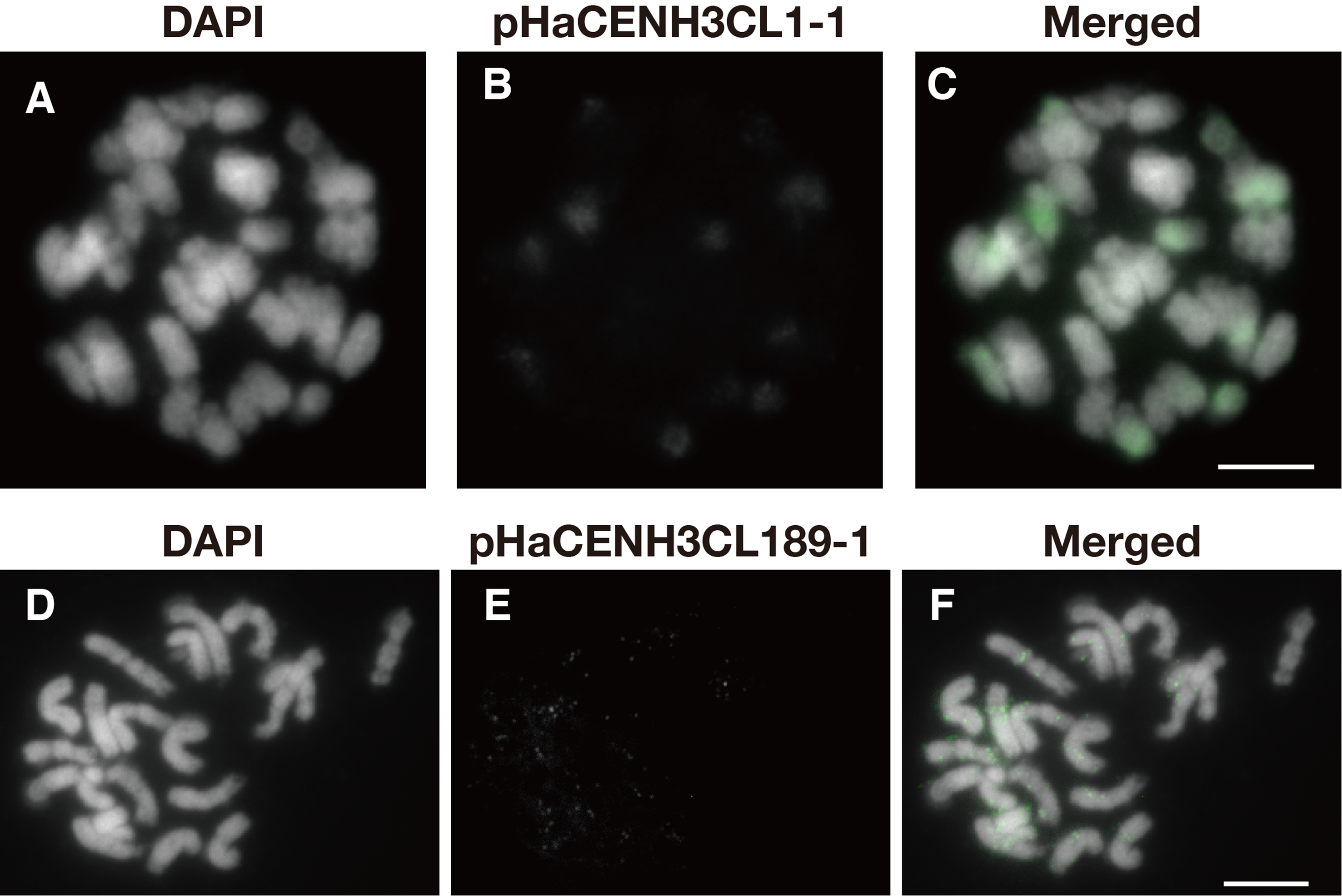

Supplement: Supplementary Image 5 — FISH using the accumulated sequence from ChIP-Seq. (A,D) DAPI-stained sunflower chromosomes. (B) FISH signals of pHaCENH3CL1-1. (E) FISH signals of pHaCENH3CL189-1. (C) A merged image of (A,B). (F) A merged image of (D,E). Scale bar, 10 μm. [file Image5.TIF]

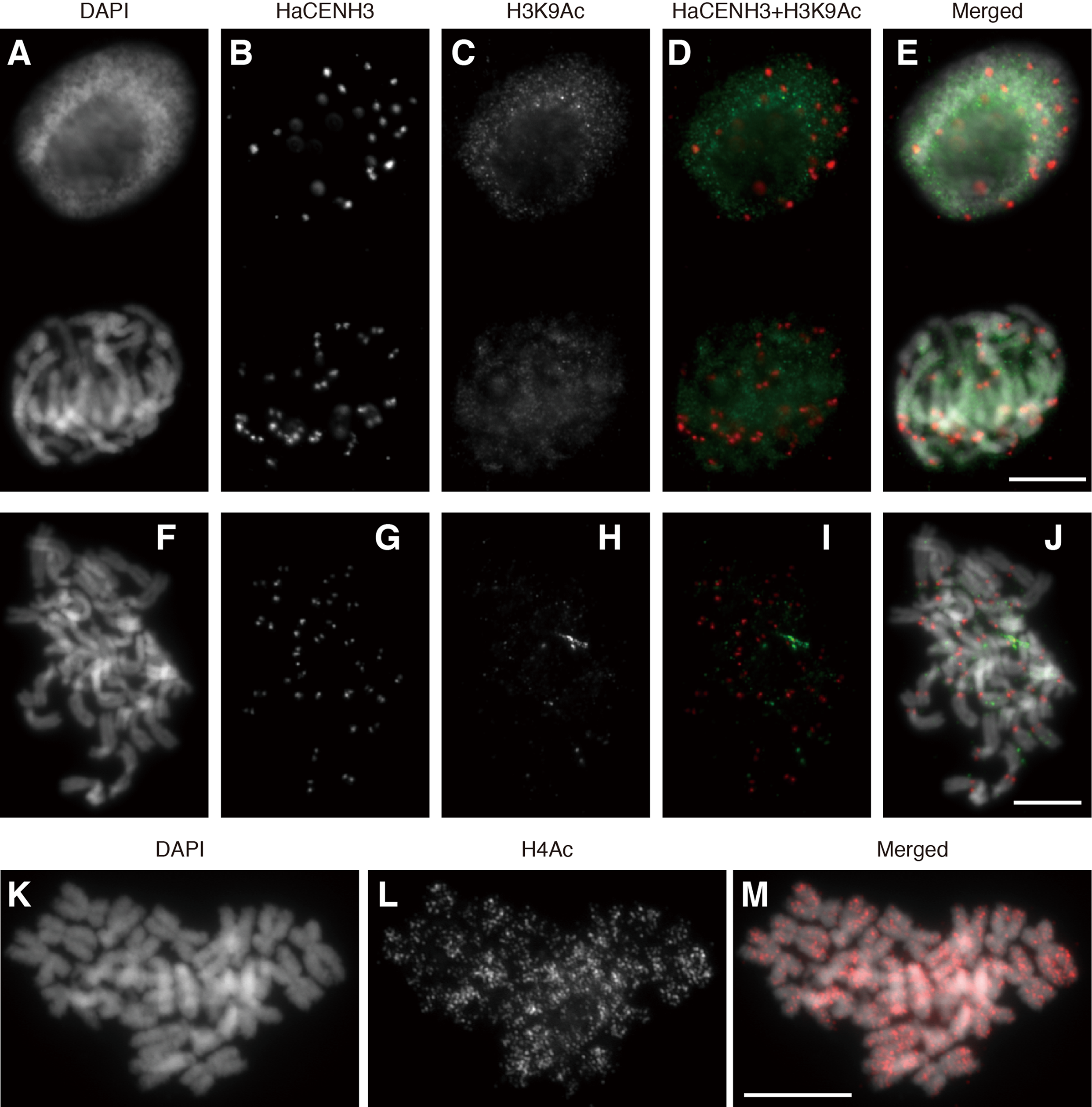

Supplement: Supplementary Image 6 — Immunostaining using the anti-HaCENH3, anti-H3K9Ac, and anti-H4Ac antibodies. (A–E) An interphase nucleus (top) and prophase chromosomes (bottom). (F–M) Metaphase chromosomes. (A,F,K) DAPI staining. (B,G) Immunosignals of anti-HaCENH3. (C,H) Immunosignals of anti-H3K9Ac. (L) Immunosignals of anti-H4Ac. (D) A merged image of (B,C). (E) A merged image of (A–C). (I) A merged image of (G,H). (J) A merged image of (F–H). (M) A merged image of (K,L). Scale bar, 10 μm. [file Image6.TIF]
