# Supplementary material for: Sunflower centromeres consist of a centromere-specific LINE and a chromosome-specific tandem repeat
Source: Front Plant Sci. 2015 Oct 31;6:912. doi: 10.3389/fpls.2015.00912 (PMC4628103; doi:10.3389/fpls.2015.00912)
Supplement: Supplementary Table 1 — Primers used in this study. [file Table1.DOC]

Supplementary Table 1. Primers used in this study

| Name | Purpose | Sequence |
| --- | --- | --- |
| HaCENH3-3RACE | 3’RACE | 5’-GTGGAGAAAACAGGAAGCCGCATAGGT |
| HaCENH3-5RACE | 5’RACE | 5’-AACTACCATGGCTGCCCTTTCTTACC |
| HaCENH3CL1-F | qPCR | 5’-GAGCGGGTCGTTGTGGA |
| HaCENH3CL1-R | qPCR | 5’-TGTAGCGGAAGCATAGTAAATAA |
| HaCENH3CL20-F | qPCR | 5’-TTGAGGGCTGGGGATAATAATACG |
| HaCENH3CL20-R | qPCR | 5’-TCCCGAATCTCTAATAACATCAAT |
| HaCENH3CL22-F | qPCR | 5’-ACTACCCACGGCTTGTCA |
| HaCENH3CL22-R | qPCR | 5’-YTGTGGGAGGATATCTGTAAAC |
| HaCENH3CL124-F | qPCR and FISH | 5’-CAAATTAAACCGAGTCTGGATGA |
| HaCENH3CL124-R | qPCR and FISH | 5’-ATGGGTGGTATtCGGATTCTG |
| HaCENH3CL289-F | qPCR | 5’-GCATCCCCCGCTCGTAACC |
| HaCENH3CL289-R | qPCR | 5’-CAAGAAGCGTGCCTGGATGA |
| HAG004N15-F | qPCR and FISH | 5’-ATGCGGATTAGATACCAAAAA |
| HAG004N15-R | qPCR and FISH | 5’-TGCCGTCACTAAACACATCC |
| HaUbi-F | qPCR | 5’-GTATCCCACCAGACCAACAGAGA |
| HaUbi-R | qPCR | 5’-AAGACGGAGAACCAAATGAAGAGT |
| HaCENH3CL1-FISH-F | FISH | 5’-ATGCCATGCTTAGAGAT |
| HaCENH3CL1-FISH-R | FISH | 5’-TGTGTCGAAAGATAAGGTTGGATT |
| HaCENH3CL20-FISH-F | FISH | 5’-GAATCACATCCGGGGGTCTC |
| HaCENH3CL20-FISH-R | FISH | 5’-ATGTGGGGCCGAAGTAGTTATG |
| HaCENH3CL189-FISH-F | FISH | 5’-TGCCGCACCAAATCACC |
| HaCENH3CL189-FISH-R | FISH | 5’-TTCCGGAGCAATACAAAGTTAGAC |
