# Supplementary material for: Sunflower centromeres consist of a centromere-specific LINE and a chromosome-specific tandem repeat
Source: Front Plant Sci. 2015 Oct 31;6:912. doi: 10.3389/fpls.2015.00912 (PMC4628103; doi:10.3389/fpls.2015.00912)
Supplement: Supplementary Table 2 — Clusters possessing an enrichment ratio (ER) higher than 2.0 in the RepeatExplorer analysis. [file Table2.DOC]

**Supplementary Table 2. Clusters possessing an enrichment ratio (ER) higher than 2.0 in the RepeatExplor**er analysis.

| Cluster name | ER | Percentage of the input | Sequence features or similar sequences |
| --- | --- | --- | --- |
| 124 | 52.0 | 0.008% | 187 bp mini sat |
| 286 | 32.7 | 0.001% | LINE-ENDO |
| 332 | 26.8 | 0.001% | LINE-ENDO and LINE-RT |
| 182 | 24.0 | 0.009% | LINE-RT and LINE-ENDO |
| 147 | 23.8 | 0.013% | LINE-RT, LINE-ENDO, Ty3-INT and Ty3-RT |
| 22 | 22.0 | 0.051% | LINE-ENDO, LINE-RT, DHH-CD1, DHH-CD2, Ty3-PROT, Ty3-RH and Ty3-RT |
| 141 | 20.8 | 0.016% | LINE-RT |
| 154 | 20.6 | 0.014% | LINE-RT, LINE-ENDO and Ty3-INT |
| 200 | 20.5 | 0.008% | LINE-RT |
| 223 | 19.9 | 0.006% | LINE-RT |
| 216 | 19.5 | 0.006% | LINE-RT and LINE-ENDO |
| 57 | 19.1 | 0.041% | LINE-ENDO, LINE-RT, DTM-CD1, Ty1-RT and Ty3-INT |
| 188 | 17.8 | 0.011% | LINE-RT and LINE-ENDO |
| 356 | 17.8 | 0.001% | LINE-RT |
| 178 | 17.7 | 0.012% | LINE-RT, LINE-ENDO and Ty3-RT |
| 172 | 17.6 | 0.013% | LINE-RT, LINE-ENDO and DTM-CD1 |
| 252 | 17.3 | 0.004% | LINE-RT |
| 20 | 16.9 | 0.068% | LINE-RT, LINE-ENDO, DTA-CD1 and Ty3-INT |
| 17 | 16.3 | 0.071% | Ty3-RT, Ty3-INT, Ty3-GAG, Ty1-INT LINE-RT, DTC-CD1 and LINE-ENDO |
| 358 | 15.8 | 0.001% | No |
| 330 | 15.5 | 0.002% | LINE-ENDO |
| 145 | 14.6 | 0.022% | LINE-RT, LINE-ENDO, DTC-CD1, Ty3-INT and Ty3-RT |
| 291 | 11.7 | 0.003% | LINE-RT and LINE-ENDO |
| 183 | 11.3 | 0.018% | LINE-RT, Ty3-INT and DTC-CD1 |
| 289 | 10.8 | 0.003% | LINE-RT |
| 335 | 6.6 | 0.003% | Ty3-INT |
| 115 | 3.1 | 0.121% | LINE-RT and LINE-ENDO |
| 247 | 2.8 | 0.022% | Ty3-INT |
| 249 | 2.6 | 0.022% | None |
| 189 | 2.5 | 0.058% | Ty3-INT, Ty3-RT, Ty3-RH, Ty3-GAG, Ty3-PROT and Ty3-CHDII |
| 214 | 2.4 | 0.040% | Ty1-GAG, Ty1-INT and Ty1-PROT |
| 100 | 2.3 | 0.173% | Ty3-INT, Ty1-INT, Ty3-CHDII and Ty3-RT |
| 359 | 2.2 | 0.007% | Ty1-PROT |
| 78 | 2.2 | 0.217% | LINE-RT, Ty1-RT and Ty3-RT |
| 13 | 2.2 | 0.440% | Ty3-INT, Ty3-RT, Ty3-RH, LINE-RT, LINE-ENDO, DHH-CD1, DTH-CD1, DTM-CD1, Ty1-RT, Ty1-RH and Ty1-INT |
| 122 | 2.1 | 0.142% | Ty1-int |
| 169 | 2.1 | 0.081% | None |
| 269 | 2.1 | 0.018% | None |
| 192 | 2.1 | 0.065% | DTM-CD1 |
| 282 | 2.0 | 0.014% | LINE-ENDO |
| 1 | 2.0 | 1.005% | Ty3-RH |
